# Conspecific mortality and resource availability drive a trait-mediated cascade by modifying sea urchin grazing behavior

**DOI:** 10.1007/s00442-026-05949-8

**Published:** 2026-08-10

**Authors:** Casey J. Sheridan, Zachary H. Randell

**Affiliations:** 1 Department of Ecology and Evolutionary Biology, University of California, Santa Cruz, Santa Cruz, CA USA; 2 Conservation Programs & Partnerships Department, Seattle Aquarium, Seattle, WA USA

**Keywords:** Trophic cascade, Alarm cues, Kairomones, Refuge use, Prey behavior

## Abstract

Predator risk effects on prey can cascade through food webs and influence ecosystem state via trait-mediated indirect effects (TMIEs). Predicting when these interactions occur and whether they produce community-level effects requires understanding the cues prey respond to, how responses vary with prey attributes and environmental context, and how these components interact. In kelp forest ecosystems, changes in sea urchin foraging behavior in response to food availability or predators can cause state shifts from forests to urchin barrens. It is less clear what cues of predation risk urchins respond to, how these cues interact with food availability, and their effects on kelp mortality. We conducted a field experiment in Monterey Bay, California using cages containing refuge habitat with live kelp (*Macrocystis pyrifera*) and purple sea urchins (*Strongylocentrotus purpuratus*) to test for separate and combined effects of a predator cue (red rock crab, *Cancer productus*), a damaged conspecific cue (crushed urchins), drift kelp, and urchin satiation on urchin exposure (i.e., use of locations exposed to predators), grazing, and kelp mortality. While predator presence did not affect urchin behavior, the damaged conspecific cue reduced urchin exposure (74%), grazing (48%), and kelp mortality (58%). Urchin satiation also decreased the number of exposed grazing urchins (96%) and consumption of live kelp (84%). Adding drift kelp nearly prevented kelp consumption regardless of the damaged conspecific cue, overriding its effect. These findings show predation risk cues can interact with resource availability to modify prey behavior, demonstrating how a trait-mediated indirect effect may cascade to alter ecosystem state.

**Supplementary Information:**

The online version contains supplementary material available at https://doi.org/10.1007/s00442-026-05949-8.

## Introduction

Prey response to predation risk can have ecosystem-wide effects via trophic cascades, where the direct effects of predators on their prey indirectly affect species in lower trophic levels. This can result in profound changes and occurs across a broad diversity of marine, freshwater, and terrestrial ecosystems (Terborgh et al. 2010; Ripple et al. 2016). Historically, the study of trophic cascades focused on top-down control with direct consumption reducing prey abundances (Hairston et al. 1960; Pace et al. 1999), which in turn indirectly influence other species, in what are defined as density-mediated indirect effects (DMIEs; Abrams et al. 1996; Peacor et al. 2020). While the importance of predators in reducing prey density is undeniable, increasing recognition is now paid to how predators can elicit a variety of non-lethal phenotypic responses in prey including changes in morphology, life history, physiology, development, or behavior (e.g., Peacor and Werner 2002; Hoverman et al. 2005; Miner et al. 2005). These changes in prey traits can then also indirectly influence additional species via trait-mediated indirect effects (TMIEs; Abrams et al. 1996; Peacor et al. 2020). Further review of classic examples of trophic cascades has revealed the previously underappreciated role of TMIEs in the strength and outcome of the cascade (Peckarsky et al. 2008). Often it is predator-induced changes in the foraging behavior of prey that alters their distribution and rates of consumption (Schmitz et al. 1997, 2004; Heithaus et al. 2009) and ultimately determines the spatial and temporal patterns and strengths of trophic cascades. The strong effects of TMIEs stem from their large impact on the per capita interaction strengths of predators on prey (Werner and Peacor 2003) and because predators can alter the phenotypes of many more prey individuals than the number directly consumed (Peacor and Werner 2002).

While it is clear TMIEs can have large effects that cascade to affect ecosystem structure and functioning, understanding how ecological and environmental contexts influence the form and strength of TMIEs remains a challenge (Wirsing et al. 2021). For example, prey typically face trade-offs between predation risk and foraging gains (e.g., Sih 1980; Holbrook and Schmitt 1988), so changes to these relative costs and benefits due to resource levels or competition can dictate the presence and magnitude of risk effects (e.g., Luttbeg et al. 2003; Bolnick and Preisser 2005; Preisser et al. 2009). Attributes of prey (e.g., size, age, or condition) and predators (e.g., hunting mode, prey preferences) may also influence prey response to predation risk (e.g., Schmitz et al. 2004; Biro et al. 2005; Matassa and Trussell 2014). Cues of predators that prey respond to (e.g. visual, auditory, chemical, tactile), also shape the strength or form of their responses (Schoeppner and Relyea 2009; Weissburg et al. 2014). Furthermore, instead of responding to cues associated with the presence of a predator, prey may instead respond to other cues of predation risk such as conspecific alarms or post-attack injury or mortality (Brown 2003; Ferrari et al. 2010). Thus, to advance our understanding of and ability to predict the strength of TMIEs and their ecosystem-wide consequences, we need to identify the forms of TMIEs (e.g., prey responses to cues), their relative importances, the factors that modify these responses (e.g., resource availability), and how these drivers interact with one another.

Kelp forests are especially appropriate ecosystems to study the complex dynamics of TMIEs because the interactions between sea urchins, the dominant grazer, and kelp have interacting sources of variation and ecosystem-wide consequences. Like terrestrial forests that lose vegetation to the forest floor, the biomass of living kelp can detach and move across the seafloor as detritus (henceforth, “drift kelp”). To lower predation risk, urchins occupy cracks and crevices where they remain inactive, feeding passively on drift kelp they can access from within this refuge. With declines in predators (Estes and Palmisano 1974; Cowen 1983; Burt et al. 2018) or resource levels in the form of drift kelp (Harrold and Reed 1985; Rennick et al. 2022), urchins shift from passive foraging on drift kelp while concealed in the reef, to active grazing upon live algae while exposed to predators on open surfaces of the reef, eventually resulting in “urchin barrens” devoid of most macroalgae (Filbee-Dexter and Scheibling 2014).

During the past decade, giant kelp (*Macrocystis pyrifera*) forests of central California experienced the regional extirpation of a sea urchin predator, the sunflower star (*Pycnopodia helianthoides)* associated with sea star wasting disease (Prentice et al. 2025) and reduced kelp productivity associated with the 2014–2016 marine heatwave. These events reduced the availability of drift kelp, increased active grazing by purple and red urchins (*Strongylocentrotus purpuratus* and *Mesocentrotus franciscanus*, respectively), decreased kelp cover, and resulted in the establishment of patchy urchin barrens (Fig. S1, Smith et al. 2021, 2024; Smith and Tinker 2022). In general, urchins switching their mode of foraging behavior from passive to active may be the key mechanism that determines ecosystem shifts to urchin barrens (Lowry and Pearse 1973; Harrold and Reed 1985), including in Monterey Bay, CA (Smith and Tinker 2022). Thus, understanding the mechanisms that modify this behavioral shift in purple urchins that determines their grazing rates on live kelp could inform kelp forest restoration and management.

Predator control of actively grazing urchins (Bernstein et al. 1981; Cowen 1983) may be caused either by consumption of exposed urchins by predators or by changes in urchin behavior. When urchins seek refuge or flee in the presence of predators such as lobsters (*Panulirus interruptus;* Matassa 2010) or sunflower stars (Duggins 1983), it can change grazing rates (TMIE) separate from a change in density (DMIE) of a local urchin population. In central California, however, *P. helianthoides* are no longer present, and otters do not forage substantially on urchins in barrens (Smith et al. 2021), raising questions whether any predators that remain in barrens can reduce urchin grazing. Urchin predators vary by region and in their effects on urchins (Eisaguirre et al. 2020). Because predators can differ in how and where they can kill urchins, each predator may generate a unique cue that influences an urchin’s behavioral response. Alternatively, the chemosensory cue of nearby predation in the form of a crushed conspecific urchin individual may be sufficient to trigger behavioral changes without the cue of a predator itself present (Tegner and Levin 1983; Vadas and Elner 2003; Watson and Estes 2011; Spyksma et al. 2017). There may also be different behavioral responses to a predator itself than to predator-induced damage or mortality (e.g., aggregation vs. movement; Tegner and Levin 1983). Furthermore, if urchins respond primarily to the chemicals released with the death of or damage to an urchin caused by predators rather than the predators themselves, then predator identity may be less important. Thus, simultaneously evaluating multiple interacting sources of variation in the strength of TMIEs will enhance our ability to predict spatial and temporal variation in the importance of TMIEs in this and other predator-prey-resource systems.

As the relative importance of food availability, predator presence, and conspecific mortality cues all have the potential to influence the predator-urchin-kelp TMIE in central California, we simultaneously evaluated all three interacting sources of variation. We used both drift kelp presence and lab manipulation of purple urchin satiation to evaluate the role of food resources in the TMIE. We also evaluated the response to the red rock crab, *Cancer productus*, a predator that is still abundant locally (see Appendix S1). Like other species of the genus *Cancer*, red rock crabs are known urchin predators that crush their prey (Low 1975; Lindberg 1985; Yamada and Boulding 1998; Steneck et al. 2013, personal observations). Because many urchin predators crush their prey, we used the crushing of a live purple urchin as a cue of predation mortality (hereafter referred to as the “damaged conspecific cue*”*) and evaluated its effect on the TMIE. Additionally, management efforts to reduce purple urchin densities often cull purple urchins via crushing, so the added behavioral effects on any nearby purple urchins are important to understand.

Here we ask the overarching question, how do prey respond to different types of risk cues? We predicted that purple urchins (hereafter referred to as “urchins”) might respond to either a predator cue (i.e., crab presence) or damaged conspecifics (i.e., crushed urchins) by reducing time spent in exposed grazing (H1a), and that these behavioral responses might alter urchin grazing rates and kelp mortality (H1b). We further predicted that the behavioral response would depend on the presence of drift kelp as an alternative resource (H2a) as would the cascading effect on kelp grazing and mortality (H2b), and on the condition (i.e., satiation state) of urchins (H3).

## Methods

To test these hypotheses we conducted subtidal field caging experiments that imitated a standardized reef habitat with live giant kelp (*M. pyrifera*) in an area exposed to urchin predators and with crevices providing refuge for urchins (Fig. S2). We then manipulated the presence of predators (*C. productus*, hereafter “crabs”), a damaged conspecific cue, and drift giant kelp to monitor how urchin grazing behavior and live kelp mortality responded to each in isolation and together.

### Experimental design

From July to September 2019, we deployed an array of 20 subtidal (5–6 m depth) cages in a sandy area adjacent to the reef at Hopkins Marine Station in Pacific Grove, California, USA (36° 37 ‘12.3” N 121° 54’ 08.2” W). Cages consisted of a 1 m x 1 m x 0.5 m rebar frame with a rigid plastic mesh floor encased in 1.5 by 1.5 cm square mesh nylon netting and covered with a layer of fine mesh fiberglass window screening to prevent small (> 1 mm) particulate matter from entering or exiting the enclosures. Each cage contained four stacks of two paving stones to simulate a crevice-like partial refuge in a rocky reef with multiple hard surfaces to secure against (Fig. S2).

Prior to each trial, we populated cages with 20 *S. purpuratus* of the most common test diameter (3–4 cm) collected from a nearby barren that had persisted for the past five years at the time of collection. Based on the site history and comparatively low gonadosomatic index (GSI = (gonad wet weight)/(urchin wet weight) * 100) of urchins collected here (barren mean GSI of 0.65; SE = 0.14 vs. kelp forest mean of 7.74; SE = 0.72), we inferred a high hunger state of these urchins. Mean natural *S. purpuratus* densities across five barren sites in the study region were 17 per m^2^ (or 18.7 *S. purpuratus* and *M. franciscanus* combined per m^2^), akin to our 20 per m^2^ experimental urchin density. We collected new urchins for each 48-hour trial and acclimated them for approximately 18 h in the cages prior to the start. After the acclimation period, we affixed one small live kelp sporophyte (~ 50 cm tall, 2–3 basal stipes) to the exposed surface in the center of each of the top four paving stones. This basic setup of paving stones with four live kelp individuals and 20 urchins in a cage was used for all experiments and was designed to imitate the foraging habitat of urchins and test whether behavior would be altered by the variables we manipulated.

### Effects of predator cue and damaged conspecific cue on urchin behavior, grazing, and live kelp mortality

#### Hypotheses

To determine the relative effects of predator and damaged conspecific cues on urchin behavior, grazing, and kelp mortality, we tested the following two hypotheses: the separate and combined effects of a predator cue and damaged conspecific cue **(**H1a) will reduce the proportion of exposed grazing urchins and (H1b) will reduce live kelp consumption and kelp mortality. To determine how long the effect of the damaged conspecific cue persists, we performed a separate experiment to evaluate the duration of urchin behavioral response (described in Appendix S3).

#### Design

**Fig. 1:**
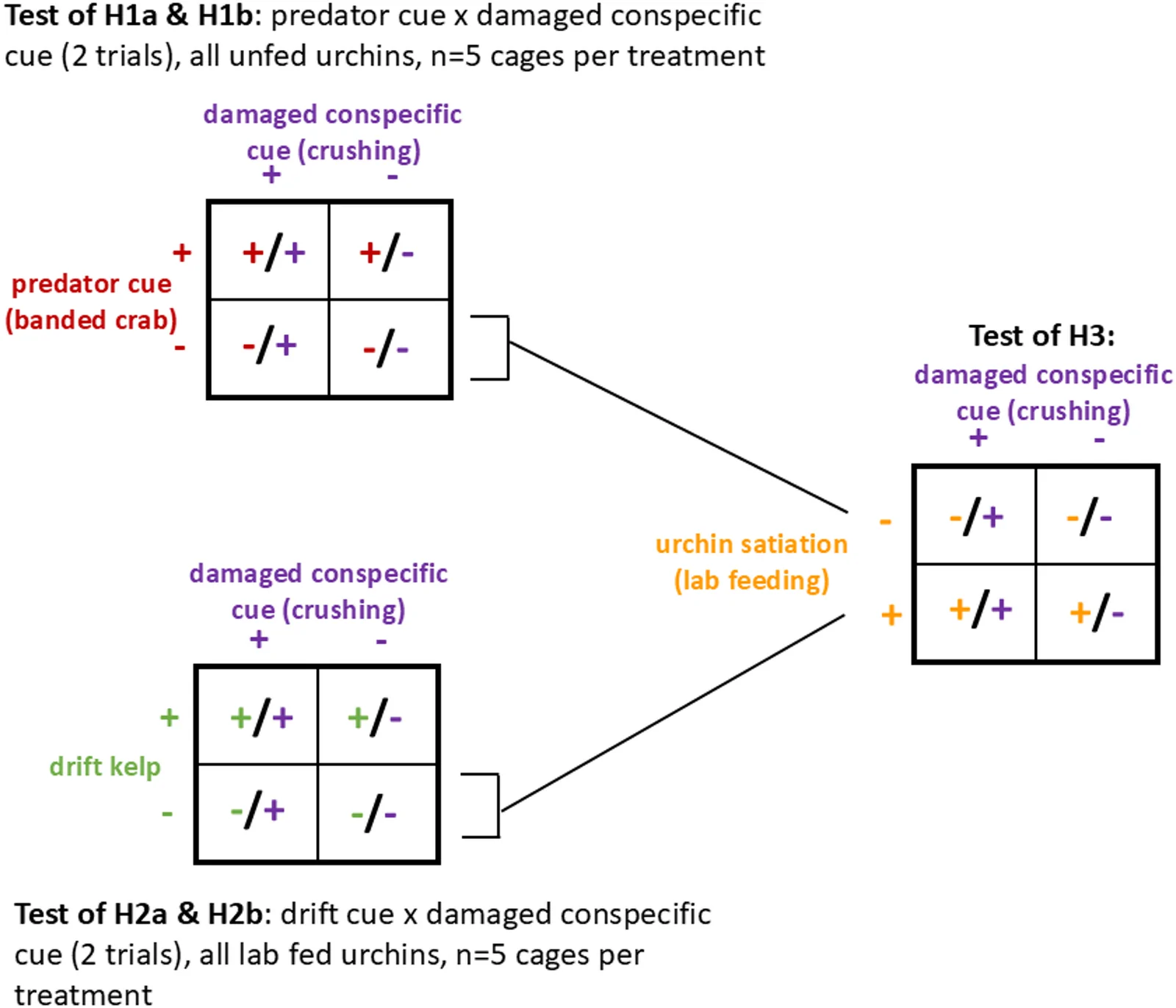
Diagrammatic overview of the three experiments conducted, the treatments in each, and the numbered hypotheses tested

To evaluate the effects of predator cues and damaged conspecific cues on urchin grazing behavior, we orthogonally crossed treatments of +/− predator and +/− crushing to produce five replicates of each combination (Fig. 1). A single crab (mean carapace width ~ 14 cm) was used as the predator in each +predator treatment, which was allowed to move freely in the cage, but its claws were closed with tape to prevent it from preying upon urchins. Thus, the predator cue potentially consisted of two parts: a chemical cue and a tactile cue from crabs physically interacting with urchins. Though crabs could physically contact urchins, there was not evidence of physical damage. For the damaged conspecific cue, two additional urchins of similar size were added to plastic mesh packets on top of two of the paving stones and crushed in place at the start of each day. We repeated this entire experimental setup with the same treatments (and crabs) randomly reallocated among cages and with new urchins in two separate 48-hour trials.

#### Sampling

In this and subsequent experiments, two of the primary response variables were the number of urchins seeking refuge and the number on the exposed top surfaces of the paving stones grazing on live kelp. Using SCUBA, divers observed and recorded the position and associations of every urchin at approximately 0.5, 3, 6, 21, 22, 25, 28, 48 h after the addition of the kelp and the application of the treatments (e.g., crushing urchins and/or adding the crabs). At each observation point we recorded the substrate of each urchin and the number of hard surfaces it contacted. We used counts of the urchins touching kelp on the exposed tops of the stones for subsequent analyses of exposed grazing urchins and urchins touching two or more surfaces as urchins “seeking refuge” (see Appendix S2 for further detail).

We measured the biomass of live kelp consumed by urchins and the mortality of kelp to assess the effect of the urchins on the kelp. We took the wet weight of kelp at the start and end of each trial (including pieces now detached from the live kelp) to estimate consumption due to grazing. This likely underestimated grazing due to potential growth of the kelp occurring during the trial. We recorded kelp mortality when urchins chewed through the basal portion of the stipes, severing the thallus from the holdfast.

#### Analysis

To assess the impact of the treatments on the proportion of exposed urchins grazing on kelp we combined data from both trials and used a repeated measures (unequal variance structure) general linear mixed model with each cage (*n* = 20) as a replicate subject and each sampling observation as the repeated measure. The mixed model used a full factorial design of our fixed effects: crush, predator, and observation time (the number of hours from the start of the experiment). Trial number was incorporated as a random effect.

To analyze live kelp consumption (i.e., urchin grazing) at the end of a trial we used a mixed model and for mortality of kelp we used a standard least squares mixed model with REML due to missing values. For both models, crush, predator, and crush*predator were used as fixed effects and trial as a random effect. All analyses described here and in the following sections used JMP Pro 17, while graphs based on these analyses used R (version 4.4.1). Effects tables from all model analyses can be found in Appendix S4. Additionally, we calculated Cohen’s d, the difference in means of the two treatments standardized by the pooled standard deviation (Cohen 1992) using the raw urchin observation and grazing data for each experiment to compare the effect sizes of treatments within and between experiments (Table 1). A Cohen’s d value of 0.2 is considered of small magnitude, 0.5 is medium, 0.8 is large, and 1.2 is very large (Sawilowsky 2009).

**Table 1:** Summary of experimental treatments, the analyses performed, and the results

Experimental treatments	Response variable (Hypothesis #)	Model type	Significant fixed effects (bold) Non-significant fixed effects (non-bold)	Main conclusions
Predator cue (crab) x damaged conspecific cue (crushing)	Proportion refuge seeking urchins (H1a)	Linear mixed model with repeated measures	Crushing (*p* < .0001), observation time *p* < .0001), crushing*observation time (*p* < .0001) predator, observation time*predator, crushing *predator, observation time *crushing*predator Cohen’s d effect size: crushing: 1.22 predator: 0.17	Crushing increased refuge-seeking and decreased exposed grazing behavior with effects that changed over time. Crushing also decreased live kelp consumption and mortality. Predator presence did not affect any response variable or interact with crushing
	Proportion exposed stone top kelp grazing urchins (H1a)	Linear mixed model with repeated measures	Crushing (*p* < .0001), observation time *p* < .0001), crushing*observation time (*p* < .0001) predator, observation time*predator, crushing *predator, observation time*crushing*predator Cohen’s d effect size: crushing: 1.89 predator: 0.09	
	Live kelp consumption (g) (H1b)	Linear mixed model	Crushing (*p* = .0002) predator, crushing*predator Cohen’s d effect size: crushing: 1.37 predator: 0.40	
	Kelp mortality (proportion) (H1b)	Linear mixed model	Crushing (*p* < .0001) predator, crushing*predator Cohen’s d effect size: crushing: 1.99 predator: 0.26	
Duration of response to damaged conspecific cue (crushing)	Proportion exposed stone top kelp grazing urchins	Linear mixed model with repeated measures	Observation time (*p* < .0001), crushing (*p* < .0001) observation time*crushing Cohen’s d effect size: crushing: 1.39	Crushing decreased exposed grazing for ~ 48 h
Damaged conspecific cue (crushing) x drift kelp	Proportion refuge seeking urchins (H2a)	Linear mixed model with repeated measures	Observation time (*p* < .0001), crushing (*p* = .0006) observation time*crushing, drift, observation time* drift, cruising* drift, observation time*crushing*drift Cohen’s d effect size: crushing: 0.38 drift: 0.05	Crushing increased refuge-seeking behavior, while drift kelp decreased exposed grazing and live kelp consumption
	Sum of exposed stone top kelp grazing urchins each day (H2a)	Generalized linear model (Poisson)	Drift (*p* < .0001), crushing period*crushing*drift (*p* = .0251) crushing period, crushing, crushing period*crushing, crushing period*drift, crushing*drift Cohen’s d effect size: crushing: 0.26 drift: 0.45	
	Live kelp consumption (g) (H2b)	Linear mixed model	Drift (*p* = .0263) crushing, crushing*drift, drift consumed Cohen’s d effect size: crushing: 0.32 drift: 0.69	
Urchin satiation state (fed/unfed) x damaged conspecific cue (crushing)	Proportion refuge seeking urchins (H3)	Linear mixed model with repeated measures	Crushing (*p* < .0001), fed (*p* < .0001), crush*fed (*p* = .0299), observation time (*p* < .0001), observation time*crushing (*p* < .0001), observation time*fed (*p* < .0001) observation time*crushing*fed Cohen’s d effect size: crushing: 0.53 feeding: 1.86	Lab feeding increased refuge-seeking behavior, decreased exposed grazing, and decreased live kelp consumption
	Number of exposed stone top kelp grazing urchins (H3)	Generalized linear mixed model (Poisson)	Observation time (*p* < .0001), crushing (*p* < .0001), fed (*p* < .0001) crushing*fed Cohen’s d effect size: crushing: 0.88 feeding: 1.62	
	Live kelp consumption (g) (H3)	Generalized linear mixed model (Poisson)	Fed (*p* = .0491), crushing (*p* < .0001) crushing*fed Cohen’s d effect size: crushing: 0.53 feeding: 1.98	

### Effects of drift kelp presence and damaged conspecific cue on urchin behavior, grazing, and live kelp mortality

#### Hypotheses

To evaluate the relative effects of drift kelp and a damaged conspecific cue on urchin behavior, grazing, and kelp mortality we tested the following two hypotheses: The separate and combined effects of drift kelp presence and a damaged conspecific cue will (H2a) reduce the number of exposed kelp grazing urchins and (H2b) reduce live kelp biomass consumed and kelp mortality.

#### Design

To evaluate the effects of resource availability and damaged conspecific cues on urchin grazing behavior, we orthogonally crossed treatments of +/− drift kelp and +/− crushing to produce five replicates of each combination (Fig. 1). The drift kelp treatment entailed adding 160 g wet weight of kelp blades cut from mature kelp individuals and torn into pieces approximately 30 cm long at the start of the trial. Crushing treatments were applied as described previously. Urchins for this experiment were again collected from an urchin barren but then brought back to lab mesocosms and fed kelp *ad libitum* for 2.5 weeks prior to the start. This period of feeding was to imitate the conditions when drift kelp might be found in a kelp forest or forest-barren interface where urchins would not be completely starved and instead might receive pulses of food.

#### Sampling

Observations and crush treatments were performed as described in the previous sampling protocols. For this experiment, we also recorded all urchins associated with drift kelp during each observation point. In addition to measuring the amount of live kelp consumed, all drift kelp pieces were weighed before and after placement in the cage to estimate drift kelp consumption. We repeated this setup with the same treatments in two separate 48-hour trials.

#### Analysis

To assess the impact of the crushing and drift kelp on the number of refuge-seeking urchins and the number of exposed kelp grazing urchins in a cage, we combined data from both trials and used mixed models. For the proportion of urchins seeking refuge, we used a general linear mixed model with repeated measures (unequal variance structure) with each cage (*n* = 20) as a replicate subject and each sampling observation as the repeated measure. We used trial as a random effect and a full factorial design of our fixed effects: crushing, drift kelp, and observation time. To analyze the number of urchins grazing on live kelp we modified this approach to deal with the many observations of zero exposed grazing urchins. Here we split each 48-hour trial into two time periods: the ~ 24 h after the first crushing at the start and the second ~ 24 h lasting from the second crushing on day two until the end of the trial. Within each period we had four observations of urchin behavior. Instead of proportions, for each of the four treatments we took the mean of the total number of exposed urchins grazing live kelp from each time period. These means were used as the response variable in a generalized linear model (Poisson distribution) with crushing, drift kelp, and crushing*drift kelp as fixed effects. To analyze live kelp consumption (i.e., grazing) we used linear mixed models with crush, drift kelp, crush*drift kelp, and drift kelp consumed (to account for differences in drift kelp consumption between cages) as fixed effects and trial as a random effect. Kelp mortality was too minimal to analyze statistically in this experiment.

### Effect of satiation state on urchin behavior and live kelp grazing

#### Hypotheses

To determine how the satiation state of urchins alters grazing behavior and the response to a damaged conspecific cue, we tested the following hypothesis: (H3) Feeding urchins will reduce the proportion of exposed grazing urchins and the effect of grazing on kelp and increase the strength of response to the damaged conspecific cue.

#### Design

The shared experimental design across experiments enabled an evaluation of the effect of the lab feeding period (with and without crushing) on urchin grazing behavior and kelp, even though a dedicated experiment was not run for this purpose. For this comparison, the non-fed urchin data came from the +crushing/-predator and -crushing/-predator treatments in the two trials of the predator cue x damaged conspecific cue experiment (i.e., excluding + predator treatments–see Fig. 1). The lab-fed urchin data comes from the +crushing/-drift kelp and -crushing/-drift kelp treatments in the two trials of the drift kelp x damaged conspecific cue experiment (i.e., excluding +drift kelp treatments). A caveat of making comparisons using this design is that the results are potentially confounded by time and varying environmental conditions.

#### Analysis

To assess the impact of the treatments on the proportion of exposed kelp-associated urchins in a cage we combined data from all four trials. To deal with the many zero values in our proportion data for lab-fed urchins, we instead used counts of exposed grazing urchins for analysis and a generalized linear mixed model (Poisson distribution). We incorporated repeated measures by using each cage as a random effect with behavioral observations nested within the cage for a given trial and treatment. Trial with feeding (+/-lab-fed) nested within was also used as a random effect. A full factorial design of crushing, feeding, and observation time were used as fixed effects for our full model. To test interactions between observation time and other fixed effects (nonhomogeneous slopes) we ran the model and sequentially removed the least significant effects one at a time. We selected the fully reduced model which included crushing, feeding, crushing*feeding, and observation time as fixed effects. To evaluate live kelp consumption at the end of the trial we used a mixed model with crushing, feeding, and crushing*feeding as fixed effects and with trial as a random effect nested within feeding.

## Results

### Effects of predator cue and damaged conspecific cue on urchin behavior, grazing, and live kelp mortality

**Fig. 2:**
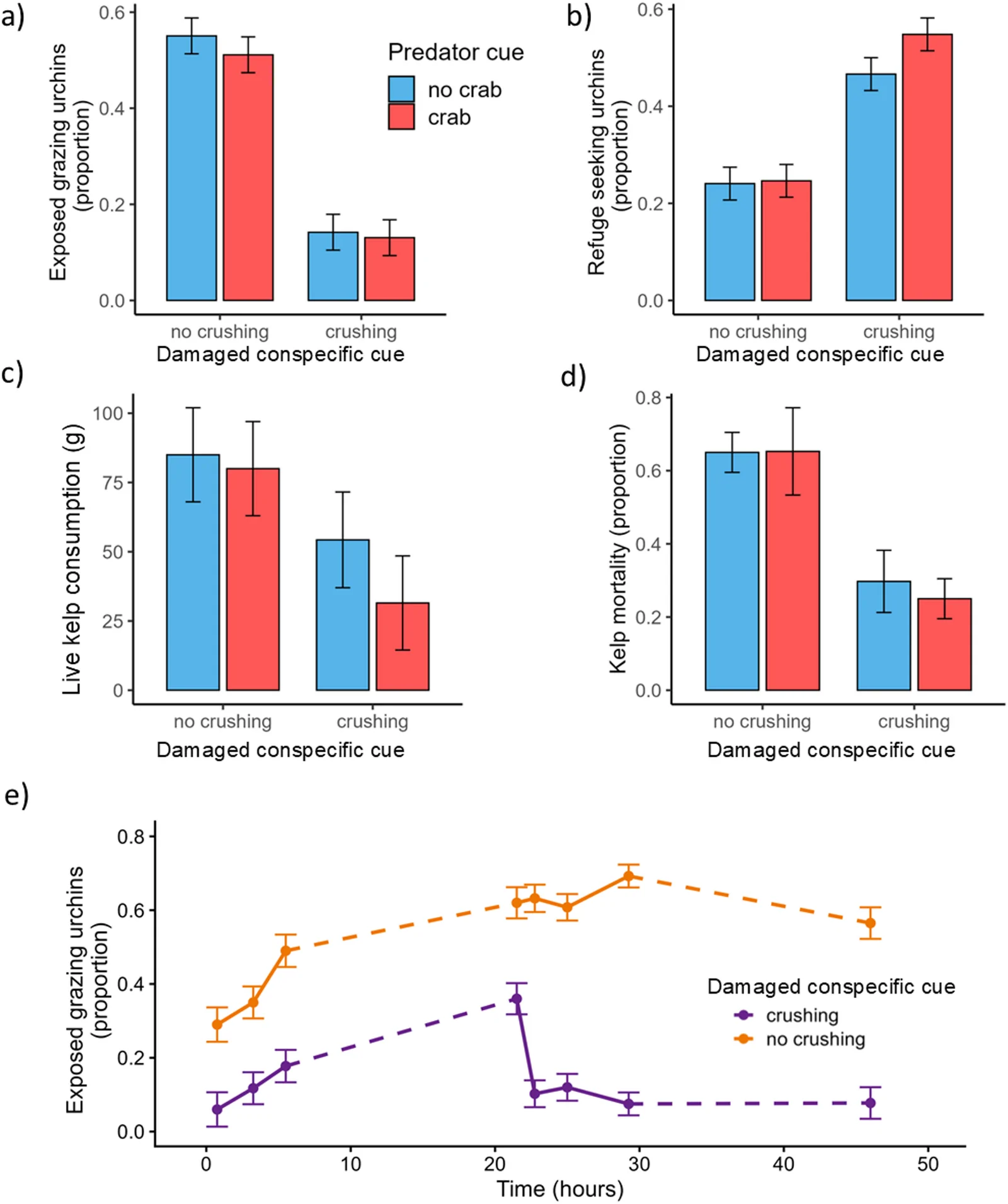
Results (least square means from the applicable model) by treatment for damaged conspecific cue (crushing) x predator cue experiment for effects on response variables of urchin behavior (**a**,** b**) and live kelp (**c**,** d**) and (**e**) time series of exposed urchins grazing on live kelp with and without crushing. Urchin behavior data includes eight observations of each cage (*n* = 20) per trial, with two 48-hour trials combined. (**a**) Mean proportion of urchins exposed on the paving stone tops and interacting with kelp in a cage under each treatment. (**b**) Mean proportion of urchins seeking refuge in a cage by treatment. (**c**) Average biomass of live kelp consumed by urchins (initial – final wet biomass in grams). (**d**) Average proportion of kelp individuals (out of four) killed by urchin grazing (severed stipes at holdfast) at the conclusion of a trial. (**e**) Time series of the mean proportion of urchins grazing on live kelp on the exposed stone tops with and without crushing over the course of a 48-hour experiment. Two urchins were added and crushed in situ at the time points indicated by the vertical blue dashed lines. The effect of the predator cue (crab presence) treatment was non-significant and is not depicted here. Dashed portions of line denoting proportion of urchins exposed and grazing on live kelp indicate longer gaps between observations resulting in greater uncertainty. All error bars are one standard error of the mean

The damaged conspecific cue significantly reduced the proportion of exposed grazing urchins (Fig. 2a) and increased the proportion of urchins seeking refuge (Fig. 2b), while predator presence did not have an effect on its own or an interaction with crushing (Table 1, Table S1, S2). Cages with crushed urchins had a lower proportion of grazing urchins than those without at each observation point (Fig. 2e), regardless of the presence or absence of the live banded crabs. Large reductions in exposed grazing urchins were observed following crushing events. There was a significant effect of observation time in the models for exposed grazing and refuge-seeking urchins that also interacted with crushing. For example, the proportion of urchins exposed and grazing increased over time without crushing, while for cages with crushing, there was an especially large decline in exposed grazing urchins following the second crushing on day two (Fig. 2e).

Overall, crushing reduced live kelp consumption and kelp mortality, while predator presence had no effect and did not interact with the crushing effect (Table 1, Table S3, S4). Live kelp consumption was reduced to nearly half in the presence of the damaged conspecific cue (Fig. 2c) and while the +crushing/+predator cue treatment combination showed the smallest amount of live kelp consumed, it was not significantly different from the +crushing/-predator cue treatment combination. Kelp in cages with crushing also experienced less than half the mortality rate seen in cages without crushing (Fig. 2d).

### Effects of drift kelp presence and damaged conspecific cue on urchin behavior, grazing rates, and live kelp mortality

**Fig. 3:**
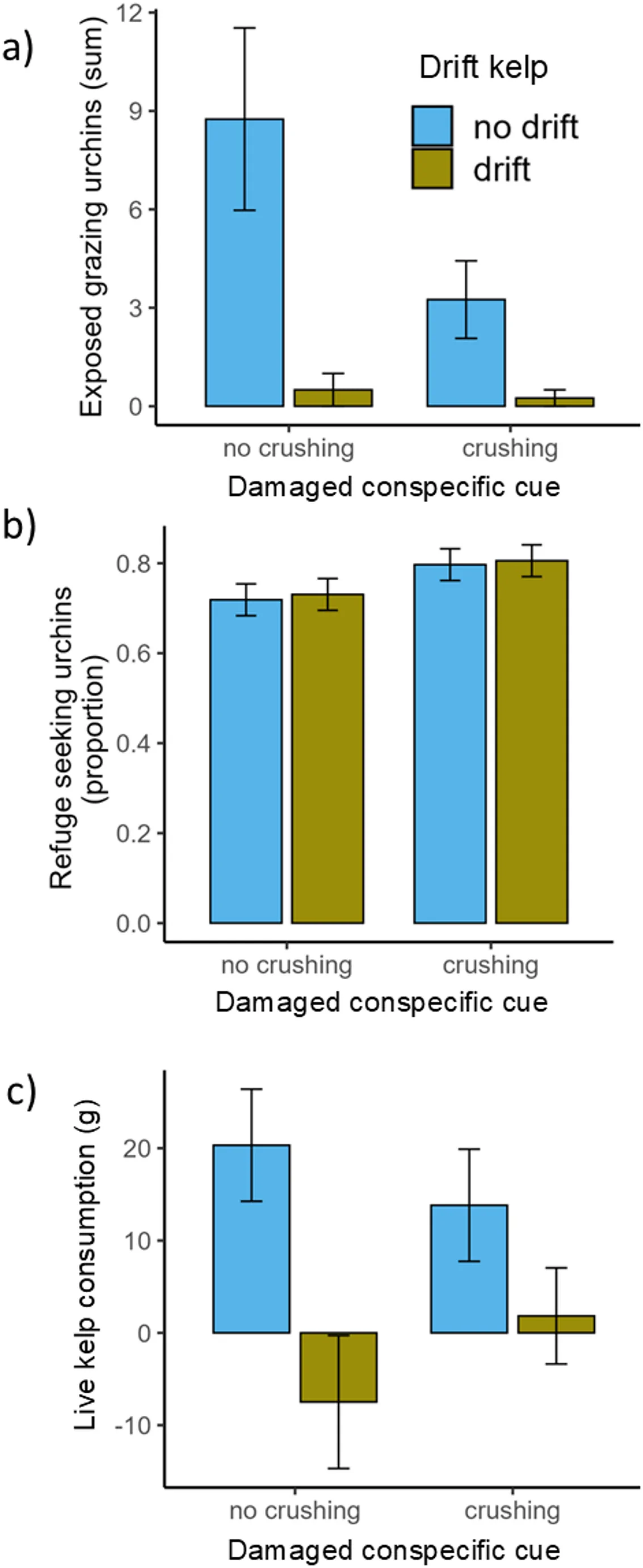
Results (least square means from the applicable model) by treatment for damaged conspecific cue x drift kelp experiment for effects on response variables of urchin behavior (**a**,** b**) and kelp (**c**). Urchin behavior data includes eight observations of each cage (*n* = 20) combined from two 48-hour trials. (**a**) Mean total number of urchins observed grazing on live kelp on the exposed stone tops under each treatment during each crushing period (i.e., day). Each cage had 20 urchins with four observations a day (totaling 80 instances of urchin behavior recorded per day), thus only a small fraction of the urchin observations recorded urchins on the grazing on live kelp on the exposed stone tops. (**b**) Mean proportion of urchins seeking refuge was high under each treatment with a significant crushing effect. (**c**) Average biomass of live kelp consumed (initial - final wet weight) by treatment. Negative live kelp consumption here is likely to indicate growth of the live kelp exceeding any losses due to grazing. All error bars are one standard error of the mean

Overall, drift kelp reduced the proportion of exposed urchins grazing on live kelp and crushing increased the proportion of refuge-seeking urchins. Drift kelp had a significant effect in reducing the average proportion of exposed urchins grazing on live kelp and the drift kelp*crushing*crushing period interaction was also significant (Table 1, Table S6). When drift kelp was available almost no urchins were exposed and grazing on live kelp. In the absence of drift kelp, the crushing treatment reduced the number of exposed grazing urchins (Fig. 3a). Still, in all treatments the total number of exposed grazing urchins observed was quite low, which precluded extensive grazing on live kelp (even the -drift kelp/-crushing only averaged around eight counts of an exposed urchin grazing across the eight observations over 48 h). The average proportion of urchins seeking refuge was high across all treatments but was significantly higher with crushing than without (Fig. 3b), while the presence of drift kelp did not have a significant effect on it (or an interaction with crushing; Table S7).

Consumption of live kelp was significantly lower in cages with than without drift kelp (Fig. 3c). Negative live kelp consumption in the presence of drift kelp indicates that live kelp growth exceeded urchin grazing. Crushing, the amount of drift kelp consumed, and the interaction of crushing*drift kelp did not have statistically significant effects on live kelp consumption (Table 1, Table S8). The crushing*drift kelp interaction was nearly significant but actually trended towards the +crushing/-drift kelp treatment having the least live kelp consumed. Kelp mortality was extremely low across all treatments during the short duration of this experiment, so we were unable to compare differences between treatments statistically. For example, in all of trial two only a single cage in the -crushing/-drift kelp treatment had any kelp mortality.

### Effects of urchin satiation on urchin behavior and live kelp consumption

**Fig. 4:**
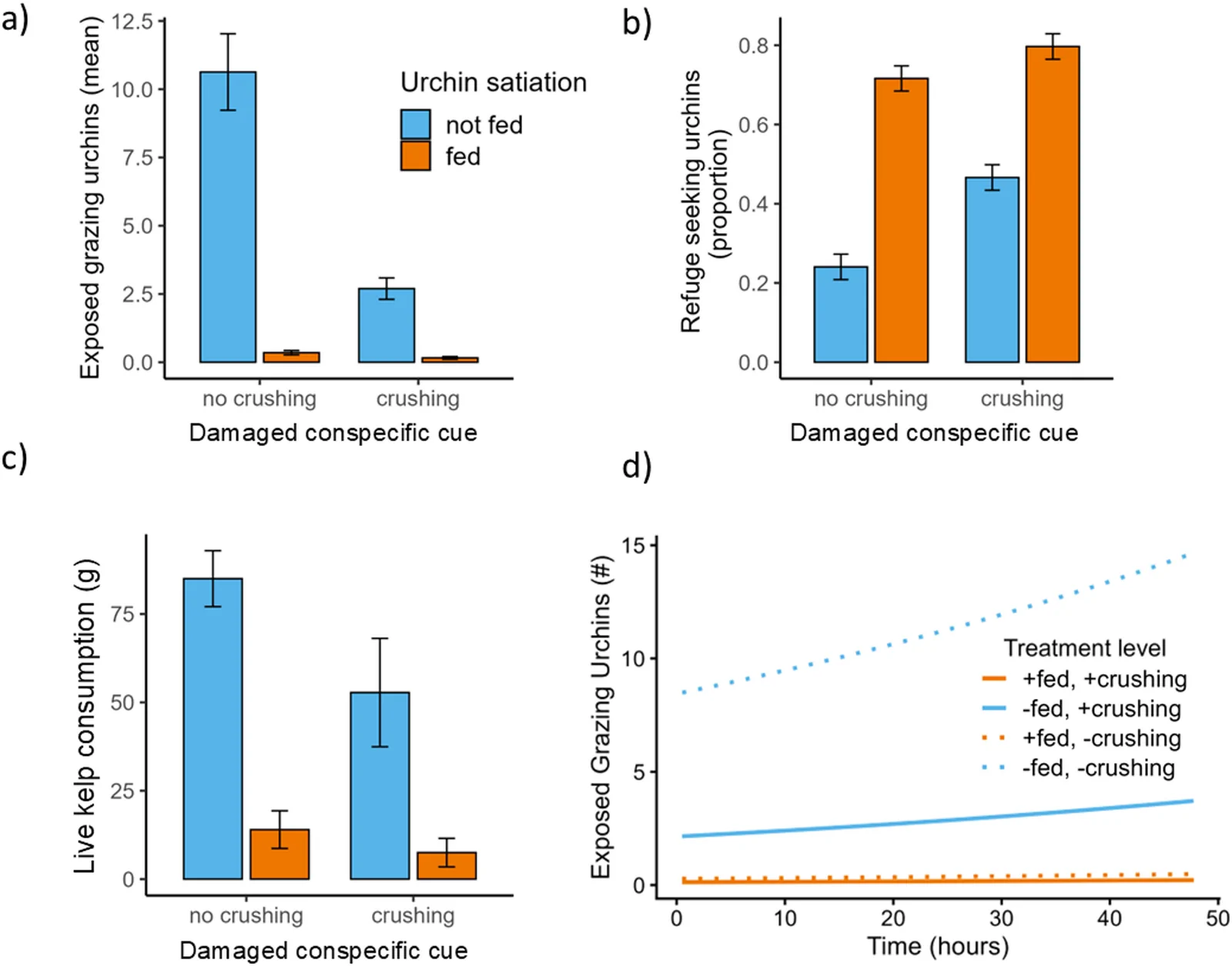
Results by treatment for damaged conspecific cue x lab feeding comparison for response variables of urchin behavior (**a**,** b**,** d**) and kelp (**c**). Results show data from four separate trials (**a**) Estimated model mean (GLMM) number of urchins grazing on live kelp (out of 20) on the stone tops for a cage under each treatment at a sampling observation. (**b**) Mean proportion of urchins seeking refuge in a cage under each treatment. (**c**) Average biomass of live kelp consumed (initial - final wet weight in grams) (**d**) Depicts the relationship between observation time and the number of exposed urchins grazing on live kelp on the stone tops based on predicted values from the model for fed (orange) and unfed urchins (blue), with (solid line) and without crushing (dotted line). All error bars are one standard error of the mean

The kelp feeding treatment (*ad libitum* for 2.5 weeks in tanks in the lab) had a strong effect on the behavior of urchins. Crushing and feeding both significantly reduced the number of exposed urchins feeding on live kelp and these effects were additive (i.e., no significant interaction; Fig. 4a; Table 1, Table S9). While the number of exposed urchins grazing on the stone tops increased over time in all treatments, there were always more non-fed than fed urchins regardless of crushing (Fig. 4d). Feeding also caused a significant increase in the number of urchins seeking refuge (Fig. 4b). Crushing increased the proportion of urchins seeking refuge as well, with a significant crushing*feeding interaction due to a stronger crushing effect on refuge-seeking behavior for non-fed urchins (Table 1, Table S10). With prior feeding, live kelp consumption was significantly lower than without feeding (Fig. 4c, Table S11). This led to minimal kelp mortality with fed urchins (only 2 of 4 kelps killed in a single cage, no mortality in all others) compared to much more extensive kelp mortality with unfed urchins (average proportion of kelps killed of 0.3125 with crushing and 0.65 without). Comparing the strength of the damaged conspecific cue across experiments (Test of H3; Fig. 1), the effect size of crushing was much greater in the unfed than the fed urchin experiments for both exposed urchin grazing behavior (Cohen’s d; 1.89 vs. 0.26; Table 1) and live kelp consumption (Cohen’s d; 1.37 vs. 0.53).

## Discussion

We found a strong predation risk effect through changes in the grazing behavior of purple urchins, which translated to reduced grazing rates on and mortality of young live kelp. The cue that induced changes in grazing behavior was the death (crushing) of conspecifics and not a cue from the predator itself, and this behavioral effect persisted for almost two days. However, the simulated longer-term effect of drift kelp resource availability (lab feeding) also had a very strong effect in reducing the number of exposed urchins, urchin grazing rates on live kelp, and kelp mortality. Exposed grazing further declined with the addition of drift kelp, and the interaction between drift kelp availability and the predation risk TMIE revealed that the presence of drift kelp negated the predation TMIE.

Although rock crabs can reduce urchin grazing rates and reduce kelp mortality directly by killing sea urchins (DMIE), we demonstrate that any potential impact of direct mortality is augmented by indirect behavioral responses of the prey. Because rock crabs crush urchins in the process of predation, as observed in companion mesocosm studies in the lab and elsewhere in the literature (Yamada and Boulding 1998), this study adds to the growing body of work demonstrating that trait-mediated effects of predation cues (rather than predator cues) on their prey can have cascading effects on lower trophic levels. To our knowledge, this is the first study showing decreased grazing and impact on live kelp by purple urchins in response to cues from conspecific mortality. On the contrary, Matassa (2010) found that purple urchins in a tank exposed to cues of crushed urchins did not reduce grazing on kelp. However, our field experiment had a simulated reef with refuge habitat and exposed habitat with kelp, and these features likely influenced urchin behavior and their responses to cues. This reinforces the importance of habitat refuge as a key factor in shaping the response of prey to predation risk (Sih and Kats 1991; Turner 1996; Trussell et al. 2006).

Urchins responded to damaged conspecific cues by moving away from the sites of crushing and tucking against the stones or cage walls instead of feeding on the exposed stone tops. This strong response to crushing in the first 24 h with an effect that persists after 43 h adds to our understanding of the temporal scale of urchin response to predation risk (Fig. S3). Subsequent crushing seemed to cause an increased response (Fig. 2e), implying that repeated predation events will prolong periods of reduced kelp mortality if predators continue to kill urchins in the area. However, with further repetition past the short duration of our trials, habituation to this cue may occur. For example, Whippo et al. (2024) found that fed purple urchin grazing rates declined when exposed to chemical cues of *P. helianthoides*, but gradually increased over time with continued exposure, suggesting habituation. Evaluating the effect of the TMIE over longer time scales and comparing the duration of the response with the rates at which predators kill urchins would also help evaluate the overall effect of crushing on grazing. For instance, predictions of theory based on temporal variation in perceived predation risk suggest infrequent, high-risk scenarios with high cue concentrations will elicit the strongest responses from prey (Lima and Bednekoff 1999) and thus could over-estimate long-term effects.

The lack of response to rock crab presence (chemosensory and tactile cues) corroborates Byrnes et al. (2006), who similarly did not detect an effect of *C. productus* presence (i.e., predator cue) on the consumption rate of kelp by purple urchins. However, our findings differ from Byrnes et al. (2006) by considering and detecting a TMIE of crabs, urchins, and kelp via the damaged conspecific cue. Our results also differ from documented urchin grazing responses to predator cues of a variety of other urchin predators. Others have detected reduced grazing activities in response to chemical cues of *P. helianthoides* (Whippo et al. 2024; Mancuso et al. 2025) and spiny lobsters (Matassa 2010). In contrast, our results suggest that crabs need to kill nearby urchins to alter purple urchin behavior and their presence alone is likely insufficient. Rock crabs are very active mobile predators, suggesting that physical encounters may also elicit urchin responses and reduce urchin foraging rates. Although we typically recorded several changes in the location of crabs during trials, we observed few of these encounters with urchins because rock crabs are largely nocturnal predators and were generally inactive during the day. The potential importance of physical encounters with crabs at night on purple urchin foraging is another direction for future research.

Our results also raise questions about predator identity effects, and the relative efficacy of evolving responses to a single cue common among predators, versus responses to multiple distinct predator cues. A recent meta-analysis found that for chemosensory cues in aquatic systems, the effects of conspecific cues were stronger than predator cues, though this pattern did not hold across other systems and sensory cue types (Jones et al. 2024). Given the diverse gauntlet of predators and potential cues that prey can face, the single cue of the death of conspecifics may be a more efficient adaptive response in contrast to a diversity of distinct predator cues. This generally aligns with predictions of strong responses to generalizable threats (based on the risk assessment hypothesis; Helfman 1989) and to increased threats (based on the threat sensitivity hypothesis; Sih et al. 2010). As in this system, some predators are highly mobile (e.g., California sheephead or sea otters), and as active and wide-ranging predators may elicit a diminished prey response to this ephemeral predator cue (Preisser et al. 2007; Wirsing et al. 2021). Prey response to a nearby damage or mortality cue may be the most effective assessment of current predation risk from such predators. However, as also exemplified in this system, some predators (e.g., lobsters, sunflower stars) elicit urchin behavioral responses while other predators (e.g., rock crabs) do not. As the specific contexts of interactions in a system can shape how prey perceive and respond to predation risk (Jones et al. 2024), more work is needed to identify the factors that determine when and why prey evolve responses to particular cues, and not others and to predict the strength and variability of TMIEs across systems.

Still, more information is needed to properly evaluate the role of rock crabs in controlling urchin grazing (despite their status as a widely distributed and fishery-targeted species). While we have witnessed rock crabs in experimental field enclosures and laboratory tanks kill 10–20 urchins from barrens with minimal gonads in a 48-hour span, the rate at which crabs feed on urchins in natural kelp forests or barrens is unknown. Nonetheless, these observations, combined with the strong behavioral response of urchins to conspecific mortality, indicate that crabs could potentially reduce urchin grazing in barrens and promote kelp forest recovery. These results are similar to findings that *P. helianthoides* consume starved urchins (Galloway et al. 2023), but contrast with the sea otter, *E. lutris nereis*, which largely do not forage on urchins in barrens in central California (e.g., Smith et al. 2021). Future studies that estimate the densities of rock crabs in kelp forests and barrens, evaluate prey preferences, the spatial scale of urchin responses relative to refuge availability, and calculate urchin consumption rates in the wild could help evaluate the full indirect effect of this mesopredator (TMIE and DMIE).

Our experiments showed that the presence of drift kelp decreased grazing on live kelp. While there was no increase in the proportion of urchins seeking refuge with drift kelp (Fig. 3b), most urchins (> 70%) in the +drift kelp treatments were in contact with drift kelp, leading to lower live kelp consumption. Thus, the effect of drift kelp in this experiment was likely primarily due to its increased accessibility as water movement redistributed pieces around the cage (mimicking the natural movement of drift kelp in the field), making this form of the resource more available than the stationary juvenile kelps. Moreover, we detected a strong interaction between drift kelp availability and the predation risk TMIE, in which the presence of drift kelp negated the predation TMIE. Results of experiments in other regions testing interactions between food availability and predator cues with kelp and urchins have found contrasting results, with effects similar to ours (Scheibling and Hamm 1991) or no effect of resource availability on the response to damaged conspecific cues (Spyksma et al. 2017). Overall, although theory often suggests TMIEs should increase in magnitude as resources increase, predicting the strength of risk effects remains complicated across systems and may depend on subtle differences in the context of the natural or experimental system (e.g., Luttbeg et al. 2003; Peacor et al. 2025).

Feeding urchins prior to a trial had a very strong effect, likely through changes in urchin satiation state, greatly reducing the number of exposed urchins feeding on kelp and almost eliminating kelp mortality. This suggests the short-term behavioral responses to move toward the chemical cue of nearby kelp (e.g., Mann et al. 1984; Parnell et al. 2017) may be less important than the longer-term effect of drift kelp presence on urchin movement, foraging behavior, and condition (satiation). With regular delivery of drift kelp, we would expect more limited movement and exposure, and greater urchin gonad volume, whereas in the absence of drift kelp, urchin would gradually deplete energy stores, reduce gonad volume, and become more active foragers. These changes in foraging behavior and condition would be expected to affect the response to predators or predation events and net vulnerability. Although the crushed conspecific cue reduced grazing rates of both fed and unfed urchins, this effect was negligible for fed urchins, which largely remained in refuges and did not graze even in the absence of crushing. In contrast, Whippo et al. (2024) found that only fed urchins responded to chemical cues of *P. helianthoides*. Still, we also recognize that our results are potentially confounded by varying environmental conditions that might have contributed to differences between the trials of fed and unfed urchins. Future studies that explicitly manipulate urchin satiation as a treatment within a trial would be a more rigorous test of this hypothesis. Additionally, though we did not have a +/− drift kelp treatment in trials with unfed urchins, drift kelp may have a stronger effect on unfed urchins that are otherwise more likely to be grazing on live kelp.

Our findings with respect to drift kelp and urchin satiation state align with the conceptual model of drift kelp availability controlling urchin grazing behavior described by Harrold and Reed (1985) and thus through drift kelp, the balance of grazing capacity and kelp production may dictate ecosystem state (Rennick et al. 2022). Randell et al. (2022) also found that sites on San Nicolas Island with high substrate complexity and thus likely higher drift kelp retention were more likely to remain stable as kelp forests over time. This is also noteworthy because urchin grazing behavior is likely to be closely linked to local drift kelp production with drift promoting passive feeding and thus enabling the continued production of drift by live kelp.

In kelp forest systems the availability of drift kelp reduces urchin consumption of live kelp not only by providing an alternative food resource, but also by shifting foraging behavior to passive feeding in cracks and crevices. This dynamic also provides a notable variation on the typical tradeoff inherent in choosing habitat to reduce predation exposure or maximize foraging. Often higher resource levels decrease refuge use (e.g., Cerri and Fraser 1983; Holbrook and Schmitt 1988; Matassa et al. 2016) due to an increase in the nonconsumptive cost of predation risk (Orrock et al. 2013). Urchins under high resource levels can receive drift kelp in refuge habitat and thus access both the preferred food and relative safety in the same location. Thus, while the structure of the refuge itself does not change (e.g., in contrast to Rozas and Odum 1988), the quality of the refuge is improved by the added resources drift kelp provides and the cost of using the refuge should be greatly lowered (e.g., Donelan et al. 2017). Crucially, instead of urchins grazing in refuge eventually depleting resources (e.g., Turner 1997), the consumption of drift kelp can help establish a positive feedback by protecting live kelp from grazing, continuing drift kelp production and maintaining resource levels. As such, this suggests the effect of resource levels can depend on its form and whether prey can access it from predator refugia.

Our demonstrated effects of a damaged conspecific cues and drift kelp availability on urchin behavior, grazing rates, and kelp mortality increase our understanding of the mechanisms contributing to the documented state shifts of kelp forests to urchin barrens and their recovery. In intact forests, the importance of damaged conspecific cues as a positive feedback mechanism for maintaining the forested state could be overshadowed by the availability of drift kelp, though their combined effects likely reinforce the positive feedback. In contrast, in barrens with the absence of drift algae, damaged conspecific cues may be more influential in determining urchin grazing rates and forest recovery, highlighting the increased importance of mesopredators in this context.

Overall, these findings lead to a deeper understanding of how TMIEs control the predator-prey-third species interaction and how behavioral changes in this interaction cascade to cause shifts in the ecosystem state. This study helps reveal how a single prey species may have evolved responses to a complex array of cues, potentially generated both from specific predators and more generally from conspecific mortality. Understanding how this hierarchy of factors including predators, resources, and prey condition interact to determine consumer behavior is important to predict how behavior can cascade to affect basal trophic levels and, potentially, even the state of an ecosystem.

## Supplementary Information

Below is the link to the electronic supplementary material.

Supplementary Material 1

## Data Availability

The datasets used and analyzed during the current study are available via Dryad at the following link: https://doi.org/10.5061/dryad.f1vhhmh3g.

## Code availability (software application or custom code)

The code used in the current study is available from the corresponding author on reasonable request.
