# Supplementary material for: Conspecific mortality and resource availability drive a trait-mediated cascade by modifying sea urchin grazing behavior
Source: Oecologia. 2026 Aug 10;208(9):114. doi: 10.1007/s00442-026-05949-8 (PMC13457239; doi:10.1007/s00442-026-05949-8)
Supplement: Supplementary file 1 [file 442_2026_5949_MOESM1_ESM.docx]

## **Electronic Supplemental Material (ESM)**

**Journal name:** *Oecologia*

**Manuscript title:** Conspecific mortality and resource availability drive a trait-mediated cascade by modifying sea urchin grazing behavior

**Authors:** Casey J. Sheridan and Zachary H. Randell

Corresponding author**:** Casey Sheridan; email: [csheridan@ucsc.edu](mailto:csheridan@ucsc.edu)

## **Appendix S1: Supplemental Study System Information**

**Fig. S1:** Temporal dynamics of giant kelp (*M. pyrifera*), macroalgal drift (measured and reported here using percent cover rather than density), purple urchins (*S. purpuratus*), and red urchins (*M. franciscanus*) from subtidal community monitoring surveys. The shaded orange region denotes the 2014-2016 marine heatwave (MHW) in the northeast Pacific. Data comes from eleven long-term monitoring sites surveyed by PISCO (Partnership for Interdisciplinary Studies of Coastal Oceans) on the interior of the Monterey Peninsula (between Otter Point and San Carlos Beach). See Malone et al. 2022 for survey design details. All sites were kelp forests prior to the MHW, while after the MHW in 2017 sites color-coded in purple transitioned to urchin barrens and sites in green persisted as kelp forests. Sites in both states saw large increases in purple and red urchins after the MHW, though these increases tended to be larger in the barrens sites. All error bars are +/- one SE of the mean.

*Estimates of red rock crab abundance in the study region*

Although abundance estimates of rock crabs are difficult to produce because of their cryptic and nocturnal behavior, we can infer high rock crab abundance in the study region from southern sea otter (*Enhydra lutris nereis*) diet composition and abundance estimates. Sea otters are voracious predators to meet their high metabolic demands (Yeates et al. 2007, Thometz et al. 2014) and rock crabs represent the second largest diet item in the Monterey study region (Tinker et al. 2019). Therefore, to support the regional population size of sea otters estimated in Smith et al. 2021 (432 +/- 123), crabs are likely to be an abundant on shallow reefs and a potentially important urchin predator in the study region.

## **Appendix S2: Supplemental Experimental Methods Detail**

## **Fig. S2** *In situ* photographs of a) a cage with mesh screening, b) The arrangement of the stones (approx. 30 cm x 30 cm x 4 cm, set up ~ 6-8 cm apart) in each cage with urchins grazing on small kelp individuals attached to exposed surfaces of the stones with garden wire, c) A large number of urchins grazing on live kelp on the stone tops, d) A crushed urchin inside of a mesh packet to standardize the location of the cue. e) Urchins consuming drift within the refuge created by the stones. All urchins were capable of fitting in this space between the stones at the same time. Photo credits: Zach Randell.

***Additional experimental methods detail:***

An urchin could seek refuge by staying in a relatively protected area and securing itself against the structure of the paving stones or the sides of the cage to wait for detached kelp as in Fig. S2e. Alternatively, an urchin could actively move around the cage and would need to occupy the most exposed region, the flat top surfaces of the paving stones, in order to graze on the basal portion of the live kelp stipes as in Fig. S2c. Though our simulated refuge might not fully prevent a predator from accessing an urchin, we still consider use of these areas of the cage to be effective in identifying urchins that are exhibiting distinct behavior.

During each period of observation for each urchin we recorded the substrate it was on (e.g., cage bottom, paving stones, cage wall, etc.) and the number of hard surfaces with which the urchin made substantial contact (i.e., more significant contact than just a spine or a tube foot grazing the surface). For example, an urchin on the cage bottom up against a paving stone would be touching two surfaces, while an urchin hunkering in the cage corner would be recorded as touching three surfaces. Urchins touching two or more surfaces were categorized as “seeking refuge” (without judging the efficacy of that refuge from a potential predator) as we considered them at the time of observation to be likely trying to hide or find protection. Urchins only touching one surface were considered more likely to be in transit or else not seeking refuge from the structure of their habitat. We also noted all urchins that were in contact with the kelp (we distinguished between live and drift kelp) as those with the bottom half of the urchin oriented over the kelp or the tube feet grabbing onto it and thus presumed these urchins were in the process of feeding. Urchins in contact with live kelp while exposed on top of the stones were the “exposed grazing urchins” used as a response variable across experiments. This more stringent definition excluded exposed urchins in other areas such as the cage walls (and the less frequent urchins that were able to access the live kelp from refuge due to the grazing of exposed urchins) whose level of reaction to the cues were more open to interpretation.

Our field cage approach had some trade-offs compared to the more typical aquarium mesocosm approaches used in many cue response experiments. The size of our enclosures and the field setting enabled us to look at a variety of factors including habitat use and grazing behavior and see the effect on perhaps the most ecologically important response— kelp mortality. The subtidal setting also provided a somewhat realistic yet standardized and controlled setup with water movement (e.g. surge) providing more natural urchin behavior and chemical cue dispersal. Still, our experimental design meant accepting a number of limitations compared to evaluating urchin grazing dynamics directly on a reef. We initially attempted to use rocks collected from nearby reef to form our rocky substrate and arranged to provide refuge habitat. We found, however, that it was not possible to observe and count urchins within the reef without taking it apart and disturbing them. It was also difficult to standardize this reef between cages with respect to the amount and quality of refuge provided. For this reason, we shifted to using the paving stones to better observe the urchins and standardize the reef between cages. The spatial scale of the cages was still relatively small considering the 1 m^2^ floor of our cages and as a result urchins could not physically flee very far from the crushing cues. Additionally, with the limited 48-hour duration of our trials, we did not allow for long-term effects like habituation or desensitization. Still, these limitations are typical of many experimental studies and field manipulations on rocky reef patches at greater spatial scales and durations any improvement in natural urchin behavior would come at the cost of less control over the manipulation. We also did not explicitly consider environmental factors such as temperature or surge, but they could alter urchin behavior and grazing rates.

**Appendix S3: Supplemental Crushing Response Duration Experiment**

*Variation in strength of the urchin behavioral response to the damaged conspecific cue over 48 hours*

To build upon our work addressing how prey respond to different types of risk cues, we conducted an additional experiment to evaluate the duration of the urchin behavioral response. Following the results of the predator cue x damaged conspecific cue experiment described previously, which only detected a response to the damaged conspecific cue, we focused here on the response to crushing.

**Design:** The trial to test the duration of the crush response had 20 cages split between two treatment levels: + damaged conspecific cue and – damaged conspecific cue. The damaged conspecific cue treatment consisted of adding and crushing two urchins as previously described in Exp 1, but only a single time at the start of the trial.

**Sampling Protocols:** The previous experiments had observations timed to track the urchin behavioral response to crushing through the period in between crushing events (~24 hours), so observations here were timed to focus on the response primarily beyond that time period to provide a more complete picture. After the initial crushing, we made the standard observations of every urchin, as previously described, at similar time intervals.

**Analysis:** To assess the impact of the crushing treatment on the proportion of urchins exposed and grazing on kelp we used a general linear mixed model with repeated measures (unequal variance structure). The model treated each cage (n=20) as a replicate subject and each sampling observation as the repeated measure. The model used crush, observation time and crush*observation time as fixed effects. We then used this model to evaluate at which observation times the crush and no crush treatments are significantly different, and to see if there is a sampling observation where the difference goes away.

**Results:** The response of urchin behavior to crushing was quite pronounced in the first 24 hours and still persisted to a lesser extent after 48 hours (Fig. S3). Urchins showed reduced exposed grazing behavior in the +crushing treatment cages (Table S5) from the onset of the trial, which was statistically significant at the 0.5 hour observation. The proportion of exposed grazing urchins increased over time both with and without crushing, but a persistent gap remained between the + and – crushing cages for most of the duration. By the end of the trial, after ~43 hours from the initial crushing the proportion of exposed grazing urchins had almost converged, and the two treatments were no longer significantly different.

**Fig. S3:** Time series showing the mean (least squares) proportion of exposed grazing urchins on the stone tops and associated with kelp at six observation periods over 48 hours. The purple line depicts the treatment level of cages (n=10) with a single crushing event at the start of the experiment (the zero-hour mark) that consisted of adding two urchins to the stone tops and crushing them in place. The orange line depicts the no crushing treatment level cages (n=10). Error bars are one standard error of the mean. Dashed portions of the lines denote longer gaps between observations resulting in greater uncertainty. Points denoted with unfilled diamond symbols are added from the previously described separate but analogous experiment (graphed in Fig. 1e) to provide a comparison of exposed grazing values from the 0.5 to 21-hour period for the same treatments.

**Appendix S4: Supplemental Analysis Model Tables**

**Table S1:** Fixed effect tests from linear mixed model with repeated measures

| **Damaged conspecific cue x predator cue**  **Response variable: proportion stone exposed grazing urchins** | | | | | |
| --- | --- | --- | --- | --- | --- |
| Source | Nparm | DFNum | DFDen | F Ratio | Prob > F |
| **observation time** | 7 | 7 | 94.9 | 13.567732 | **<.0001** |
| **crushing** | 1 | 1 | 36.7 | 112.01507 | **<.0001** |
| **observation time*crushing** | 7 | 7 | 94.9 | 12.994529 | **<.0001** |
| predator | 1 | 1 | 36.7 | 0.4610919 | 0.5014 |
| observation time*predator | 7 | 7 | 94.9 | 1.1517816 | 0.338 |
| crushing*predator | 1 | 1 | 36.7 | 0.142398 | 0.7081 |
| observation time*crushing*predator | 7 | 7 | 94.9 | 1.6187648 | 0.1394 |

**Table S2:** Fixed effect tests from linear mixed model with repeated measures

| **Damaged conspecific cue x predator cue**  **Response variable: proportion refuge seeking urchins** | | | | | |
| --- | --- | --- | --- | --- | --- |
| Source | Nparm | DFNum | DFDen | F Ratio | Prob > F |
| **observation time** | 7 | 7 | 96.7 | 24.05851495 | **<.0001** |
| **crushing** | 1 | 1 | 35.7 | 60.90257647 | **<.0001** |
| **observation time*crushing** | 7 | 7 | 96.7 | 10.71131996 | **<.0001** |
| predator | 1 | 1 | 35.7 | 1.680701883 | 0.2031 |
| observation time*predator | 7 | 7 | 96.7 | 1.141863096 | 0.3437 |
| crushing*predator | 1 | 1 | 35.7 | 1.269369238 | 0.2674 |
| observation time*crushing*predator | 7 | 7 | 96.7 | 1.661900749 | 0.1275 |

**Table S3:** Fixed effect tests from linear mixed model

| **Damaged conspecific cue x predator cue**  **Response variable: live kelp consumed** | | | | | |
| --- | --- | --- | --- | --- | --- |
| Source | Nparm | DFNum | DFDen | F Ratio | Prob > F |
| **crushing** | 1 | 1 | 34 | 17.652149 | **0.0002** |
| predator | 1 | 1 | 34 | 2.1702696 | 0.1499 |
| crushing*predator | 1 | 1 | 34 | 0.8889553 | 0.3524 |

**Table S4**: Fixed effect tests from linear mixed model

| **Damaged conspecific cue x predator cue**  **Response variable: kelp mortality** | | | | | |
| --- | --- | --- | --- | --- | --- |
| Source | Nparm | DF | DFDen | F Ratio | Prob > F |
| **crushing** | 1 | 1 | 30.72 | 28.1436 | **<.0001** |
| predator | 1 | 1 | 30.72 | 0.0987 | 0.7555 |
| crushing*predator | 1 | 1 | 10.12 | 0.0587 | 0.8134 |

**Table S5**: Fixed effect tests from linear mixed model

| **Duration of response to damaged conspecific cue**  **Response variable: proportion stone top exposed grazing urchins** | | | | | |
| --- | --- | --- | --- | --- | --- |
| Source | Nparm | DFNum | DFDen | F Ratio | Prob > F |
| observation time (hours)*crushing | 5 | 5 | 46.8 | 1.8110305 | 0.1291 |
| **observation time (hours)** | 5 | 5 | 46.8 | 18.654857 | **<.0001** |
| **crushing** | 1 | 1 | 92.8 | 83.966056 | **<.0001** |

**Table S6:** Fixed effect tests from generalized linear model (Poisson distribution)

| **Drift kelp x damaged conspecific cue**  **Response variable: sum of number of stone top exposed grazing urchins** | | | |
| --- | --- | --- | --- |
| Source | DF | L-R ChiSquare | Prob>ChiSq |
| crushing period | 1 | 7.31E-09 | 0.9999 |
| crushing | 1 | 1.46E-07 | 0.9997 |
| crushing period*crushing | 1 | 2.5285936 | 0.1118 |
| **drift** | 1 | 40.401347 | **<.0001** |
| crushing period*drift | 1 | 1.01E-08 | 0.9999 |
| crush*drift | 1 | 3.00E-08 | 0.9999 |
| **crushing period*crushing*drift** | 1 | 5.0176552 | **0.0251** |

**Table S7:** Fixed effect tests from linear mixed model with repeated measures

| **Drift kelp x damaged conspecific cue**  **Response variable: proportion refuge seeking urchins** | | | | | |
| --- | --- | --- | --- | --- | --- |
| Source | Nparm | DFNum | DFDen | F Ratio | Prob > F |
| **observation time** | 7 | 7 | 116 | 4.8374584 | **<.0001** |
| **crushing** | 1 | 1 | 248.8 | 12.175556 | **0.0006** |
| observation time*crushing | 7 | 7 | 116 | 0.6838143 | 0.6855 |
| drift | 1 | 1 | 248.8 | 0.2208943 | 0.6388 |
| observation time*drift | 7 | 7 | 116 | 0.2087575 | 0.9829 |
| crushing*drift | 1 | 1 | 248.8 | 0.005071 | 0.9433 |
| observation time*crushing*drift | 7 | 7 | 116 | 0.5224994 | 0.816 |

**Table S8:** Fixed effect tests from linear mixed model

| **Drift kelp x damaged conspecific cue**  **Response variable: live kelp consumed** | | | | | |
| --- | --- | --- | --- | --- | --- |
| Source | Nparm | DFNum | DFDen | F Ratio | Prob > F |
| crushing | 1 | 1 | 34.8 | 0.1236558 | 0.7272 |
| **drift** | 1 | 1 | 30.1 | 5.4605146 | **0.0263** |
| crushing*drift | 1 | 1 | 34.8 | 3.900475 | 0.0563 |
| drift consumed | 1 | 1 | 25.6 | 2.7079524 | 0.1121 |

**Table S9**: Fixed effect tests from generalized linear mixed model (Poisson distribution)

| **Urchin satiation state x damaged conspecific cue**  **Response variable: number of stone top exposed grazing urchins** | | | | | |
| --- | --- | --- | --- | --- | --- |
| Source | Nparm | DFNum | DFDen | F Ratio | Prob > F |
| **observation time** | 1 | 1 | 305 | 33.291048 | **<.0001** |
| **crushing** | 1 | 1 | 38.8 | 19.073905 | **<.0001** |
| **fed** | 1 | 1 | 25 | 296.79744 | **<.0001** |
| crush*fed | 1 | 1 | 38.8 | 1.3880843 | 0.2459 |

**Table S10:** Fixed effect tests from linear mixed model

| **Urchin satiation state x damaged conspecific cue**  **Response variable: proportion refuge seeking urchins** | | | | | |
| --- | --- | --- | --- | --- | --- |
| Source | Nparm | DFNum | DFDen | F Ratio | Prob > F |
| **observation time** | 7 | 7 | 98 | 7.2828888 | **<.0001** |
| **crushing** | 1 | 1 | 36.6 | 22.79245 | **<.0001** |
| **observation time*crushing** | 7 | 7 | 98 | 7.3946008 | **<.0001** |
| **fed** | 1 | 1 | 36.6 | 157.91992 | **<.0001** |
| **observation time*fed** | 7 | 7 | 98 | 5.4416092 | **<.0001** |
| **crushing*fed** | 1 | 1 | 36.6 | 5.1057077 | **0.0299** |
| observation time*crushing*fed | 7 | 7 | 98 | 1.9466936 | 0.0702 |

**Table S11:** Fixed effect tests from generalized linear mixed model (Poisson) with negative values set to zero

| **Urchin satiation state x damaged conspecific cue**  **Response variable: live kelp consumed** | | | | | |
| --- | --- | --- | --- | --- | --- |
| Source | Nparm | DFNum | DFDen | F Ratio | Prob > F |
| **fed** | 1 | 1 | 2 | 18.919987 | **0.0491** |
| **crushing** | 1 | 1 | 35 | 54.826198 | **<.0001** |
| fed*crushing | 1 | 1 | 35 | 1.5934734 | 0.2152 |

**Supplemental References**

Malone, DP, Davis, K, Lonhart, SI, Parsons‐Field, A, Caselle, JE and Carr, MH (2022) Large‐scale, multidecade monitoring data from kelp forest ecosystems in California and Oregon (USA). Ecology103(5): e3630. [**https://doi.org/10.1002/ecy.3630**](https://doi.org/10.1002/ecy.3630)

Smith JG, Tomoleoni J, Staedler M, Lyon S, Fujii J, Tinker MT (2021) Behavioral responses across a mosaic of ecosystem states restructure a sea otter–urchin trophic cascade. Proc Natl Acad Sci USA 118:e2012493118

Thometz, NM, Tinker, MT, Staedler, MM, Mayer, KA and Williams, TM (2014) Energetic demands of immature sea otters from birth to weaning: implications for maternal costs, reproductive behavior and population-level trends. Journal of Experimental Biology, 217(12), pp.2053-2061.

Tinker MT, Tomoleoni JA, Weitzman BP, Staedler M, Jessup D, Murray MJ, Miller M, Burgess T, Bowen L, Miles AK, Thometz N (2019) Southern sea otter (*Enhydra lutris nereis*) population biology at Big Sur and Monterey, California--Investigating the consequences of resource abundance and anthropogenic stressors for sea otter recovery. U.S. Geological Survey Open-File Report 2019–1022, 225 p., https://doi.org/10.3133/ofr20191022.

Yeates, LC, Williams, TM and Fink, TL (2007) Diving and foraging energetics of the smallest marine mammal, the sea otter (*Enhydra lutris*). Journal of Experimental Biology, 210(11), pp.1960-1970.
